# Immunophenotyping of cerebrospinal fluid cells in virus-associated neurologic diseases

**DOI:** 10.1186/1742-4690-12-S1-P86

**Published:** 2015-08-28

**Authors:** Yoshimi Enose-Akahata, Raya Massoud, Breanna Caruso, Joan Ohayon, Bridgette Jeanne Billioux, Gloria von Geldern, Bryan Smith, Avindra Nath, Steven Jacobson

**Affiliations:** 1 Viral Immunology Section, Neuroimmunology Branch, National Institute of Neurological Disorders and Stroke, National Institutes of Health, Bethesda, MD, USA; 2 Section of Infections of the Nervous System, National Institute of Neurological Disorders and Stroke, National Institutes of Health, Bethesda, MD, USA

## 

Various inflammatory neurologic diseases are associated with viral infections. These agents may cause direct damage of infected cells associated with immunological alterations such as chronic activation, immunodeficiency and infiltration of inflammatory cells into the central nervous system that underlie the pathogenesis of inflammatory neurologic diseases. Therefore, characterization of local immune responses that are associated with the inflammatory milieu may provide evidence or pathogenic “signature” of an immunopathogenic process in virus-associated neurologic diseases. Multicolor flow cytometry is a powerful technology to provide a large amount of cellular information for each sample and allow identification and characterization of novel cell subsets, even rare cell population. Using the technique, we characterized the cellular immunophenotypes (CD4+ and CD8+ T cells, B cells, monocytes and NK cells) in cerebrospinal fluid (CSF) and blood of patients with virus-associated neurologic diseases including HTLV-1 associated myelopathy/tropical spastic paraparesis (HAM/TSP; n=18), HIV adequately treated with antiretroviral drugs (n=28), multiple sclerosis (MS; n=7) and progressive multifocal leukoencephalopathy (PML; n=8) compared to healthy volunteers (n=8). CD4+ and CD8+ T cells were significantly altered among the groups, and the CD4/CD8 ratio reduced in HIV, HAM/TSP and a subset of PML, but elevated in MS. In patients with HAM/TSP, activated CD4+ T cells were significantly increased in the CSF as well as the blood and correlated with HTLV-1 proviral DNA loads. HAM/TSP patients had an increased frequency of effector CD4+ and CD8+ T cells in the blood, but not in the CSF. Antibody secreting B cells were elevated in the CSF of patients with HAM/TSP, MS and PML and were significantly correlated with intrathecal HTLV-1 Gag-specific antibody responses in HAM/TSP patients. Immunophenotyping of CSF cells may reflect immune pathology in inflammatory neurologic diseases and can serve to highlight differential diagnoses that may lead to a better understanding of disease pathogenesis and clinical treatment.

